# Ethical values in college education: a mixed-methods pilot study to assess health sciences students’ perceptions

**DOI:** 10.1186/s12909-018-1396-7

**Published:** 2018-12-04

**Authors:** Asunción Hernando, Ignacio Diez-Vega, Marta Lopez del Hierro, Nieves Martínez-Alsina, Raquel Diaz-Meco, Maria José Busto, Noa Lola Martiañez, Gustavo González-Cuevas

**Affiliations:** 1 Faculty of Biomedical and Health Sciences, Universidad Europea de Madrid, Madrid, Spain; 2 Faculty of Sport Sciences, Universidad Europea de Madrid, Madrid, Spain; 3 Universidad Internacional de la Rioja, Madrid, Spain; 4 Centro Universitario La Salle, Madrid, Spain

**Keywords:** Ethical values, Ethical competence, Bioethics, Ethical learning

## Abstract

**Background:**

**S**ociety demands a university education grounded on ethical principles. Education in ethics values is responsibility of universities but will not be viable unless also adopted by directly responsible agents, the teachers who work with the students. For this reason, our primary research objective was to conduct an in-depth analysis of how Health Sciences students self-perceive the ethical dimension.

**Methods:**

A mixed research methodology with two phases, qualitative and quantitative, allowed us to address our research question from two complementary viewpoints. Conversational interviews were conducted in an intentional and purposive sample to identify a wide range of discursive representations. A questionnaire was designed based on previous studies and the topics of qualitative research. The response format for the questionnaire followed a Likert scale and modulators such as sex, age, degree and the score of a social desirability test were examined.

**Results:**

After 24 conversational interviews, three main thematic blocks (coinciding with the three subscales of the questionnaire) were identified: “attitudes for harmony in human relations”, “construction of the self” and “rules and regulations”. A total of 246 students completed a questionnaire with 39 items. The total scores ranged from 93 to 152 points, with an average score of 122.72 ± 10.64 points. Responsibility, the basic rules of education and respect were perceived as the two most important values, whereas solidarity and social participation as the least important. Results showed a significant positive linear correlation between total score on the questionnaire and age and social desirability. Age was also a significant predictor for the total score and the subscale score “rules and regulations”. The students´ responses seemed to be conditioned by the degree of social desirability that they present.

**Conclusions:**

The ad-hoc questionnaire captured the maintenance of high ethical values in our college undergraduate students, which may be directly related to enhanced social desirability. The scores obtained on the questionnaire were correlated with the students’ age, which may indicate that values might tend to acquire progressively more importance as students grow older. Further research is warranted to delve deeper on the determinants of professionalism and ethical decision-making in college students.

**Electronic supplementary material:**

The online version of this article (10.1186/s12909-018-1396-7) contains supplementary material, which is available to authorized users.

## Background

The model of society that is currently considered democratic implies that its citizens accept a moral minimum based on human rights, which tend to be reflected in a country’s laws. Today, our universities are undoubtedly also in need of educating responsible citizens to make an ethical commitment to the social environment [1–3].

The dynamic, enterprising and transformative nature of educational research in the European Higher Education Area (EHEA), particularly the degrees related to Health Sciences, has generated the need for new teaching intervention proposals to promote the training and integration of competencies in academic and health contexts. The aim is to improve the quality of educational processes in order to improve the quality of care provided by future health professionals.

Currently, universities train and evaluate according to competencies based on the requirements established in legislation regarding university degrees. Universities strive to ensure the quality of the results of learning based on competencies [4] and deepen the training development, also based on student competencies. For many years and coinciding with changes in curriculum that are required for the new degrees, ethical training has been a component of the Health Sciences degree programs in universities. College teaching is not limited to transmitting knowledge, and often, transmission of knowledge does not contribute to ethical development. Thus, the moral dimension of college teaching should be required as a complement to knowledge [5, 6].

Indeed, universities are facing a changing educational model that affects various objectives and methodologies. Since the early 1990s, academia has sought to change from providing an education based on content to an education based on competencies. Such education involves teaching students how to know and how to be appropriate in a given context in addition to acquiring knowledge [7].

In this line, different authors [8, 9] have argued that a curriculum to implement an educational model based on competencies must integrate competencies for lifelong learning, for living together and for life in society. Life in society encompasses the capacity to decide and act with critical judgment, addresses values and social and cultural rules, promotes transversal axes enabling the individual to act with respect toward others, embrace diversity, and combat racism and discrimination.

The years spent at the university cannot serve only to develop scientific and humanistic knowledge to a high level but should also be an experience of life, of ethical learning and of moral personality development. The university provides a sense of process and globalization to one’s education primarily through the set of interactions that occur between teacher and student [10–13].

Furthermore, it is important to note that the university education of the twenty-first century has evolved from being only based on discipline to being also based on values. Education in civil ethics values should be the responsibility of universities but will not be viable unless also adopted by directly responsible agents, the teachers who work with the students [14]. As the team of teachers who present this research, we have accepted this responsibility and are aware of the need to identify tools that will allow us to know our students better and motivate them.

Health Sciences students should know the values that are accepted in their society and reflect on which are more important to them. Only by this way, these future health care providers will be open to the human moral dimension, to their own moral dimension and to the moral dimension of patients, and they will be able to initiate a process of personal searching to become good professionals.

Some ethical values are more universal for any professional, while others are more related to those professional linked to the care and protection of life. In this sense, different authors focus their works on researching which values are what Health students consider the most important for their professional life. Thus, we find that respect, honesty, responsibility, justice or empathy, among others, are considered necessary in the profile of the professional who yearn to become [15–20].

The work of García-Huidobro et al. [21] shows us the perception that medical students have about the values that a doctor should practice, highlighting: humility, altruism, attitude of service, integrity, empathy, willingness to help, acceptance of diversity, responsibility, respect, ask help and advice to others, learning and continuous improvement, leadership, assertiveness and active listening.

In reference to Physiotherapy students, Amer Cuenca and Martínez Gramage [22] investigate the ethical values that should guide the professional behavior of a physiotherapist, highlighting: patient welfare, help to others, patient autonomy and justice.

Regarding to patients, the perception that they have on the most relevant ethical aspects in the health professionals can help us to assess if they coincide with those estimated by the students or, on the contrary, we need to strengthen them throughout the university educational process. Different authors collect in their researches that patients consider some attitudes as fundamental, such as delivery, listening, responsibility, respect and empathy [23–25].

We can deduce that patient care should be of a higher quality and more humane, with an adequate training of Health students. If teaching methods were coordinated along the transversal content and competencies, the process would be easier, allowing us to provide more experiential learning [16].

During preclinical training, it is not sufficient to introduce theoretical content related to ethics into the curriculum; students should begin to develop thoughtful and increased awareness habits with regard to fundamental values in the clinical relationship [26]. As some authors have previously warned, the human and educational experiences of the student may inhibit their moral development [27], and we know that one of the most important tasks of the teacher is to motivate students to acquire the habit of constantly incorporating essential values into their activities, based on the model of a professional that meets the demands of the era and of our society [28]. Surprising discrepancies between the values prioritized by the institution and those prioritized by students have been observed in some studies, at the beginning of their studies as much as at the end [18]. Such results should lead us to examine how the educational process is being presented and the origin of these discrepancies.

The development of the ethical commitment of a Health Sciences student is a long road that continues throughout his professional life. During this journey, teachers and tutors can support the search and soften the discomfort produced by uncertainty but should never replace or manipulate deliberation.

In 2007, a relevant study was conducted by the Spanish Secretary of State for Universities and Research with the objective of developing an ethical profile of Spanish university students. The study explored students’ attitudes in three basic dimensions of moral personality: construction of the self, living together and socio-moral reflection [29]. Social sciences and engineering students were the primary participants in this study; thus, it was important to us to know and analyze these aspects in Health Sciences students in the different degree programs in which the members of our research group taught.

As teachers who want to accompany the process of the formation of our students, we must learn more about how they live in the ethical dimension, and this search has been the main objective of our study. The specific objectives of this study have been:

- To determine the ethical values that are most important for students of Health Sciences degrees.
- To analyze whether the perception of ethical values among students is dependent on variables such as type of degree, age or sex.

A tool was designed and implemented in college students with the aim of exploring the perception of their ethical values. A mixed research methodology with two phases, qualitative and quantitative, was used to address the objectives of our study from two complementary points of view.

## Methods

### Phase 1. Qualitative study to design a questionnaire

The study was approved by the Research Committee of Health Sciences of the University, and the confidentiality and privacy of all personal data was in full compliance with the Spanish Law 15/1999 on Protection of Personal Data.

Before designing the questionnaire, the authors regarded as important to analyze the students’ perceptions of ethical values. The first stage of the study was carried out with an intentional or purposive sample with the researchers’ students who belonged to health degree programs in Medicine and Physical Therapy (simple or double degree in Physical Therapy and Physical Activity and Sport Science).

Collaboration was requested to students with a high level of participation in the classroom but also to those who did not usually participate. In order to identify a wide range of discursive representations, sex and degree were the main variables chosen for the selection of the sample. Too, in order to collect as much meaningful information as possible about their perceptions and experiences, the authors took into account, the two different Health science degrees.

The sample included was comprised of 24 Health Sciences undergraduate students at the European University of Madrid. These were first-, second-, and third-year students, 12 enrolled in degree programs in Medicine and 12 in the Physical Therapy degree. The mean age was 20 years old, with equal sex ratio. Semi-structured discussions were conducted until the speech was saturated. This enable us to ensure that all views were represented.

The open interviews were conducted by three researchers who had previously agreed on the interview strategy as a measure to avoid bias. Researches used the same motivating questions with an established average time between 15 and 20 min. To facilitate dialogue, students answered the following opening question: Now that you are an undergraduate student, which aspects, related to ethical values, do you think are more relevant for you? Students answered themselves spontaneously, and their comments and ideas were collected.

The data analysis was done following the model proposed by Taylor and Bogan [30], in four steps: data preparation, discovery of emerging issues, coding of the data and writing of the report presenting the findings of the study. So, the analysis followed a systematic model of collection-analysis-collection-analysis until discourse saturation was reached. Discourse content was analyzed to code overt content by topics or words and latent content by searching for meaning in paragraphs and the general context. Coding was performed manually by three independent researchers. In successive readings, the identified codes were grouped to generate themes and subthemes until all Information was represented. Triangulation, refered to the use of multiple methods or data sources and observers during our research, has allowed the convergence of information and also ensures a comprehensive understanding of the subject .

So, the information was codified in three thematic blocks similar to those used in the study of Buxarrais MR, Esteban F and Mellen T [20], which subsequently served as a guide to design a questionnaire. The items were built to address the most frequent issues derived from the interviews and three main thematic blocks were identified and used to categorize the qualitative data. Items included information regarding both the theoretical concepts and the students’ attitudes toward real situations. We searched for familiar and easy-to-identify examples by students.. The research team was divided into three groups to create questions for each thematic block. The authors carried out several meetings to reach consensus in content, meaning and format of these questions.

The 39 questions that composed the questionnaire are included in Additional file 1 and the Table 2. Thirteen questions collect the perception of the students on aspects related to attitudes for harmony in human relations (subscale A in the quantitative analysis): 2, 11, 12, 16, 17, 18, 28, 29, 31, 33, 34, 36 and 38. The questions related to the construction of the self (subscale B) are 1, 4,6, 7, 20, 22, 23, 25, 27, 30, 35, 37, and 39. Finally, thirteen questions collect aspects related to the rules (subscale C): 3, 5, 8, 9, 10, 13, 14, 15, 19, 21, 24, 26 and 32.

### Phase 2. Descriptive study of the ethical values explored in the questionnaire

Our sample comprised students enrolled in courses taught by the researchers, as follows:

- Second-year medical students were enrolled in the courses “Introduction to Clinical Practice” and “Epidemiology and Applied Biostatistics.”
- Third-year students in Medicine enrolled in the course “Physiopathology and Semiology.”
- First-year students in Physical Therapy and the double degree in Physical Therapy and Physical Activity and Sport Science enrolled in the courses “Physical Therapy” and “Manual Therapy.”
- Second-year students in the double degree in Physical Therapy and Physical Activity and Sport Science enrolled in the course “Data Analyses.”

The main variables of the study are the 39 items of the questionnaire collected in Additional file 1 and were described according to the obtained score pertaining to each item as well as the total score of the questionnaire. The response format for the questionnaire followed a Likert scale with scores ranging from 1 to 4 (1 = totally disagree; 2 = somewhat disagree; 3 = somewhat agree; 4 = totally agree). Possible total results in the questionnaire vary from 39 to 156 points.

In addition, moderators such as sex, age, degree and the score of a social desirability test were examined. To investigate whether our test responses were conditioned by social desirability, the Marlowe and Crowne’s social desirability scale was used [31]. This test is widely used to measure the tendency of people to answer questions in a way that will be viewed favorably by others, and thereby to establish here discriminant validity for our test [32].

The statistical analyses were carried out with the statistical software SPSS version 20. First, internal consistency reliability for the questionnaire globally and its subscales was evaluated by Cronbach’s alpha (α). The validity was justified by a group of experts in ethical values, who assessed the meaning of the questions independently and in their connection with the three dimensions explored. The descriptive statistics used were absolute frequencies, percentages, means and standard deviations (SD). To analyze whether distributions of students would differ by degrees and sex, the Chi-square test was employed. To determine statistical significant differences in global scores for the questionnaire as a function of degree, sex and academic year, t-tests for independent groups or ANOVAs were used. Finally, to study what variables (i.e, age, degree, academic year, sex, and social desirability) predict changes in the scores of the ethical values’ questionnaire, multiple regression analyses were performed. Parametric assumptions were checked before running any statistical analyses.

Significance level was *p* < .05.

## Results

### Phase 1. Qualitative study to design a questionnaire

We identified three main thematic blocks in the conversational interviews, referring to students’ perception of ethical values (see Table 1):

- Attitudes for harmony in human relations
- Construction of the self
- Rules and regulations

**Table 1:** Themes identified in the interviews of the qualitative phase

Attitudes for harmony in human relations	
Respect others	General attitude toward others
▪ *The material, their work* ▪ *Their wishes, their value* ▪ *Not to disturb* ▪ *Not to impose* ▪ *Respect others’ opinions* ▪ *Be responsible to classmates and teachers* ▪ *Environment and nature* ▪ *Not to waste the money that parents invest*	□ *Assistance* □ *Confidentiality* □ *Empathy, courtesy, listening, patience* □ *Solidarity, generosity, fairness* □ *Neutrality, justice* □ *Sensitivity to complicated cases* □ *Not to have prejudices*
Construction of the self	
▪ *Feeling good about yourself and others* ▪ *The self begins from childhood* ▪ *Self-esteem* ▪ *Self-respect* ▪ *Self-improvement* ▪ *Responsibility for oneself* ▪ *Emotional balance* ▪ *Effort-be hard-working*	▪ *Constancy* ▪ *Focus on what is important* ▪ *Organization* ▪ *Study* ▪ *Feel good for helping* ▪ *Meet the expectations of classmates and teachers* ▪ *Respond to what they ask you*
Rules and regulations	
▪ *Common standards* ▪ *Rights and duties* ▪ *What is right* ▪ *Principles and values* ▪ *Justice* ▪ *Maintain your values and respect those of others.*	▪ *Do the right thing* ▪ *Experienced since childhood* ▪ *Standards of behavior* ▪ *What is right* ▪ *Encourage the dynamics of the class* ▪ *Participate*

In the interviews, the students discussed respect and general attitudes toward others in the context of living together. The attitude that appeared most frequently in the conversations was respect, including fundamental respect of others’ opinions and values and encompassing both classmates and teachers. The participants explicitly mentioned the importance of respecting the environment and classroom materials. Values related to attitudes toward others emerged, specifically referring to relations with their groups. The students referred to the importance of behaviors that involve helping others, being generous or polite, and solidarity and sensitivity to what they called “complicated cases”. The students also indicated that empathy, listening and patience in relationships are considered important in their environment.

In the majority of the interviews, ethical values appeared to be focused on the students themselves, such as descriptions involving self-esteem and their capacity for self-improvement. In this sense, the students commented that ethical values have to do with feeling good and with emotional balance. Other aspects were related to their education and to being responsible in their studies, which were related to the possibility of being trained from a personal and professional point of view and giving importance in some cases to constancy and organizational capacity. This is the point of what an activity regarding the solidarity of personal growth can be, considering the importance of the feeling of self-realization.

There is an important component of what for the students was associated with ethics and standards. Students focused on what is right, principles, rights and duties. They also mentioned justice and current norms, including those norms learned in childhood. The importance of these norms in their current environment of the university and in their classes was reflected in their conversations, making specific reference to the value of participation and the classes’ dynamics.

### Phase 2. Descriptive study of the ethical values explored in the questionnaire

The questionnaire was completed by 246 students, 111 (45.1%) were men, 133 (54.1%) were women, and 2 (0.8%) were omissions. In terms of degrees, there were 168 medical students (68.3%), 31 Physical Therapy students (12.6%), and 47 Physical Therapy and Physical Activity and Sport Science students (19.1%).

An analysis by degree and sex showed a significant relation between the studied degree and sex of students (*p* < 0.01). Notably, the highest proportion of women, 68,6%, were pursuing a medical degree, whereas more men were represented in the double degree of Physical Therapy and Physical Activity and Sport Science, 84,78%.

In terms of age, participants did not exhibit statistically significant differences when compared by sex and degree (*p* > 0.05). The average age of the men was 20.26 (± 2.28) and that of the women was 20.71 (± 3.16).

Table 2 presents the average results obtained for each of the 39 items regarding ethical values. The global score of the questionnaire ranged from 93 to 152 points, and the overall score of the questionnaire was 122.72 ± 10.64 points.

**Table 2:** Questionnaire designed to explore the ethical values in the Health Sciences with the scores obtained

#	Item	Mean	SD
1	I am responsible for my actions.	3.78	0.47
2.	After a conversation, I can change my point of view.	3.26	0.66
3.	I know the standards of the university and my degree.	3.08	0.81
4.	My manner of acting with others is consistent with my ideas.	3.54	0.54
5.	I respect my environment, I do not litter, and I do not deface urban surfaces.	3.47	0.74
6.	When I fail a subject, I can assess what I can do to improve.	3.48	0.64
7.	I like to be updated when there is important news to reflect on what has occurred.	3.12	0.71
8.	If a teacher makes fun of a co-worker, I let my coordinator/tutor/delegate know about it.	2.45	0.93
9.	When someone is speaking, I do not generally interrupt, and I wait for him/her to finish.	3.20	0.74
10.	It seems bad to me when someone insults or interrupts a classmate or teacher.	3.72	0.55
11.	I turn to dialogue as a strategy to address conflicts.	3.61	0.57
12.	I have an open attitude and close relationships with my colleagues.	3.37	0.71
13.	I use trash bins appropriate to each type of waste.	3.05	0.94
14.	When I use computers at the university, I turn them off when I finish using them.	3.37	0.91
15.	I take special care with the material provided to me by the university (laboratories, classrooms, stretchers, tables, etc.).	3.59	0.56
16.	I am aware that consensus/agreement is not always reached through dialogue.	2,74	0.97
17.	If a friend becomes angry with me, I reflect on his reaction to try to understand him/her.	3.26	0.71
18.	I know how to be in someone else’s shoes.	3.48	0.63
19.	I introduce myself or participate in the election of delegates of courses.	1.46	0.85
20.	If a teacher suspends me unjustly, I know how to control my anger.	2.46	0.92
21.	I give up my seat on the bus to elderly, pregnant or disabled persons.	3.75	0.49
22.	I am able to defend my opinion about political or religious news although my colleagues may think otherwise.	3.50	0.66
23.	When a group of colleagues unfairly criticizes a friend of mine, I position myself in his/her favor.	3.49	0.67
24.	It is important to have a course delegate to speak to teachers and the university.	3.04	0.97
25.	I know how to organize my study and leisure hours.	2.92	0.82
26.	When a colleague speaks to me during a teacher’s explanation, I ask him/her to let me pay attention.	2.59	0.83
27.	I base my opinions on reasoned arguments.	3.48	0.52
28.	I favor a good group environment in my work team.	3.52	0.56
29.	I participated in a group or organization of a social or political nature.	1.60	0.96
30.	I know how to explain my opinions when a topic of discussion emerges in my group of friends.	3.43	0.63
31.	When a colleague thinks differently from me, I attempt to respect his/her opinion.	3.40	0.66
32.	When a course starts, I make sure to read the program and the standards.	2.33	0.97
33.	I show my disapproval of unfair treatment to a person of a different race.	3.55	0.71
34.	I relate to people of different religious beliefs, respecting their convictions.	3.62	0.61
35.	I recognize good arguments even if they do not coincide with my own.	3.63	0.53
36.	If there is a conflict between colleagues, I insist that we listen to one another and reach an agreement.	3.39	0.65
37.	I read the news at least once a week.	3.19	0.92
38.	I am involved in a volunteer activity.	2.03	1.10
39.	I reuse products that I consume as much as possible.	2.76	0.95

**Fig. 1:**
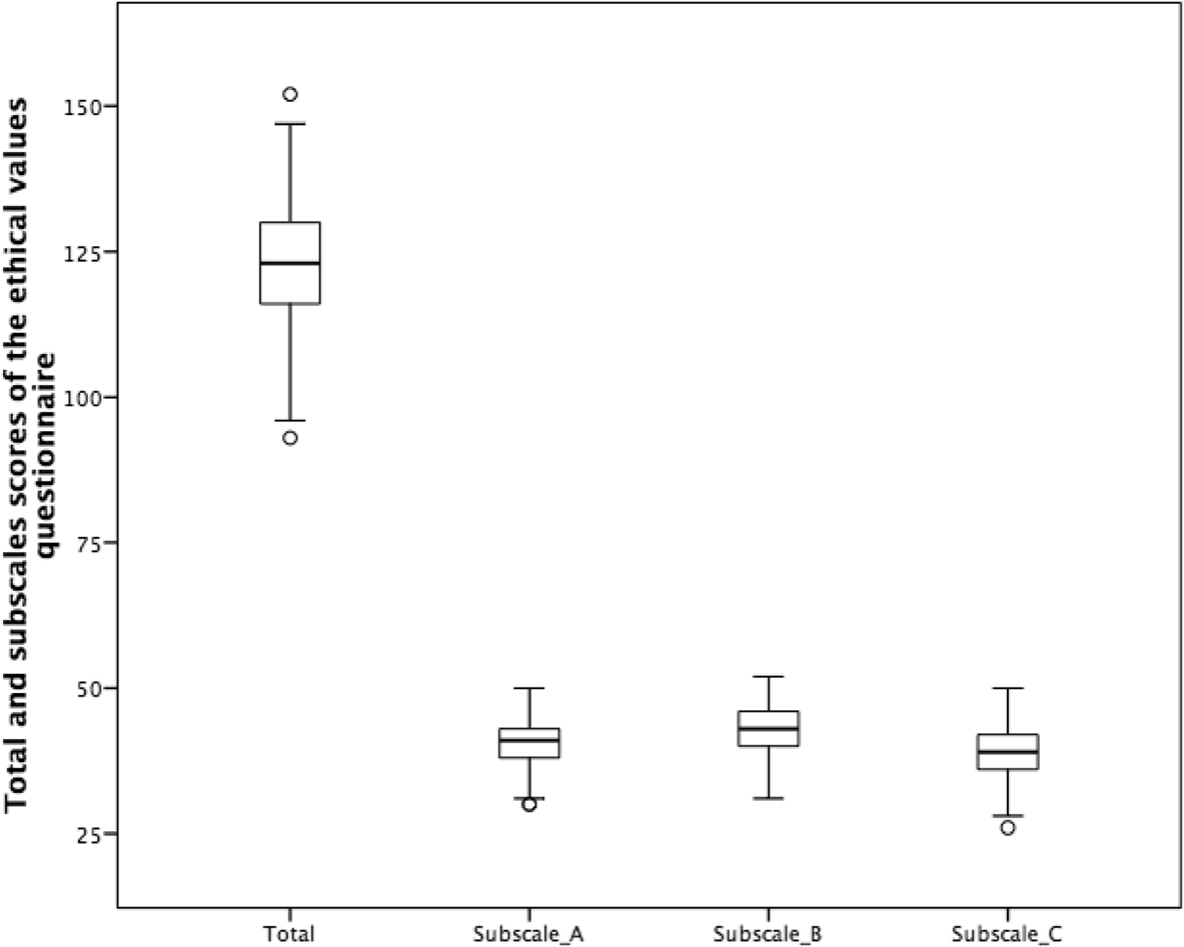
Quartiles of the total and subscale scores of the ethical values questionnaire

The study of the internal consistency of the global questionnaire of ethical values revealed a high level of reliability (α = 0.83). For subscale A (Attitudes for harmony in human relations), α = 0.62, for subscale B (Construction of the self), α = 0.68, and for subscale C (Rules and regulations), α = 0.68. The internal consistency of the questionnaire of social desirability was α = 0.67. The scores distribution of the questionnaire divided by quartiles can be observed in Fig. 1 in a box diagram.

In Table 2, it can be seen that three questionnaire items stood out as having a mean score higher than 3.7 (maximum = 4). These items were related to responsibility (item #1) and the basic rules of education and respect (items #10 and #21). Other items had also a high mean score, higher than 3.5; for example, in relation to thinking and acting with coherence (item #4), promoting dialogue as a strategy in conflict resolution (item #11), favoring a good atmosphere in groups (item #28), fostering diversity and interculturality (item #33), and the ability to recognize good arguments different from yours (item #35). Finally, the items with the lowest mean scores, lower than 2.1, were related to volunteering (item #38) and participation in the educational community (item #19), or in social or political organizations (item #29).

Global average scores of the questionnaire indicated no statistically significant differences with which to compare by degree, by sex and by degree and sex (*p* > 0.05). These data are presented in Table 3.

**Table 3:** Total and subscales scores (average) ± (standard deviation) in the questionnaire of ethical values

	Medicine	Physiotherapy	Sports/physical therapy	
Men				
Total	122.38 ± 9.66	124.85 ± 10.72	120.56 ± 10.88	122.19 ± 10.31
Subscale A	41.00 ± 4.09	40.15 ± 4.45	39.92 ± 4.21	40.47 ± 4.19
Subscale B	43.42 ± 3.43	43.15 ± 4.13	42.36 ± 4.44	43.00 ± 3.93
Subscale C	37.96 ± 4.88	41.55 ± 3.47	38.28 ± 4.52	39.45 ± 4.82
Women				
Total	123.46 ± 11.38	120.09 ± 8.78	124.57 ± 6.60	123.24 ± 10.98
Subscale A	41.23 ± 4.14	39.55 ± 4.32	42.00 ± 2.71	41.13 ± 4.10
Subscale B	42.87 ± 4.42	40.18 ± 3.71	43.14 ± 3.80	42.66 ± 4.37
Subscale C	39.37 ± 4.99	40.36 ± 3.80	39.43 ± 3.31	39.45 ± 4.82
	123.13 ± 10.86	123.16 ± 10.19	121.17 ± 10.38	

Relationships between the variables “age” and “social desirability” and the overall score obtained on the questionnaire of ethical values were explored. A linear, direct, low and significant correlation between age and the overall score of the questionnaire of ethical values was identified (*r* = 0.26; *p* < 0.001), indicating that older students scored higher than younger students.

We also observed a significant correlation between the total score of the questionnaire of ethical values and the total score of the test of social desirability (*r* = 0.33; *p* < 0.001), which appears to indicate that the students´ responses on the ethics questionnaire can be somewhat conditioned by the degree of social desirability that the students present.

As it can be seen in Table 4, the multiple regression analyses showed as a significant predictor of changes in the total and subscales scores of the questionnaire of ethical values the variable social desirability, which might indicate that the students´ responses on the ethics questionnaire can be somewhat conditioned by the degree of social desirability that the students present. Also, age was also a significant predictor for the total score and the subscale C score in the ethics questionnaire, indicating that older students scored higher than younger students. Finally, the type of degree also predicted changes in scores in the subscale A.

**Table 4:** Multiple regression coefficients

	Total	Subscale A	Subscale B	Subscale C
Age	0.20**	0.11	0.12	0.26**
Sex	**−**0.09	−0.06	−0.16	−0.01
Degree	− 0.11	− 0.20*	− 0.16	0.08
Year	0.07	0.02	0.11	0.05
Social Desirability	−0.31*	− 0.22**	− 0.16*	− 0.39**

Note: Dependent variables: Total and subscales scores of the ethical questionnaire. Significant standardized B coefficients are in boldface; *p < 0.05, **p < 0.01

## Discussion

A mixed methodology was used in the present study because currently, increasingly more researchers in the field of education endorse this method for conducting an in-depth analysis of the phenomenon studied [33–36]. Qualitative and quantitative components were flexibly combined in all of the research stages to develop a joint discussion that may allow a broader view of the analyzed situation.

Educational ethnography has been considered to be a qualitative method of research because it focuses on exploring what occurs on a daily basis in the educational field by providing meaningful data as descriptively as possible, allowing that data to be interpreted, understood and utilized in the most appropriate manner [37]. Thus, researchers should focus on the various educational scenarios in which the interactions, values, activities and expectations of all participants occur. Bisquerra [37] considered that this type of design of research is considerably more flexible and may be adapted to the context of the application, establishing an action plan that approaches the object of interest. Thus, we may develop a good rapport with and an adequate degree of involvement by participants using strategies that allow us to collect meaningful information.

We consider that the qualitative approach has allowed us to begin working with students on the ethical values from their own experience, which enabled identification of the most significant themes. Some educators consider that education is the process of incorporating values into one’s own existence, and it appears logical for college educators to work with the values that the student has [38–41].

It is assumed that throughout the students’ primary and secondary education, they will have incorporated the values that compose their moral personality, and in their university experience, they should acquire the skills that allow them to define their personal purpose in life [14, 42–44].

As a component of lifelong learning, the role of the university is prioritized as a place in which values and attitudes are not only acquired but also modified by experiences and interactions with teachers and peers [11, 45]. Here, we emphasize that the scores obtained on the questionnaire were correlated with the students’ age, which may indicate that during the years lived in the university, values acquired more importance. These data coincided with those of other university studies [6, 46].

The interviews that were conducted in the qualitative phase of the study were conducted by three professors, who agreed that the first reaction of the students was one of bewilderment. It was striking that some of the students had difficulty beginning a response to the questions they were asked, which was interpreted as an attitude of surprise in the young people because the subject matter was unusual in the university context. In a study conducted on Italian students of medicine and nursing, participants considered values to be essential to their formation but these values were uncommon in their learning process [16].

The analysis of our subjects led us to this same conclusion, that ethics are not sufficiently present in the formative process of our students. We can improve the development of ethics if we incorporate activities that integrate the analysis of and reflection on these values. We understand and agree in this regard with the recommendation of Grootenboer [47] and Morales et al. [11], who noted that today’s teachers could attempt to rethink university practices without losing the opportunity to develop the ability to allow significant learning from an ethical and citizen’s point of view.

Regarding the questionnaire, there were some interesting aspects. For instance, three of the most interesting questions, with a score higher than 3.7 (out of 4), were related to responsibility and to the basic standards of education and respect. Several studies conducted with Health Sciences students show that respect is considered the most important value [15, 48, 49]. In these studies, unlike ours, students give less importance to responsibility as a value, with a lower score than the one given to respect. In other studies, like ours, responsibility and respect obtain similar scores [18, 20].

Several issues scored approximately 3.5, such as the coherence between one’s manner of acting and one’s ideas, using dialogue as a strategy in conflict resolution, favoring a good atmosphere in their groups, aspects of interculturalism, and the ability to recognize good arguments although they are different from one’s own.

In general, total test scores were quite high, which also occurred in other studies [16, 46]. The aspect with the highest mean was related to the norms of moral obligation, which may explain the results. Therefore, and following the recommendations of Grieve and McSwiggan [50], we wanted to include a test of social desirability in our study. The internal consistency was similar to that reported in the literature [51–55]. A positive correlation was observed between the scores of the two questionnaires, which confirmed that their answers to the questions regarding moral norms were conditioned by the students, assuming a certain level of overlap.

Another interesting aspect of our analysis is that there were no differences in the averages of the questionnaire scores by academic years. This may be explained by the high scores obtained, which could imply a ceiling effect of the global scores. In this aspect, it should be noted that there is no homogeneity in what was contributed by the different studies. Montilva et al. [56] do not find significant differences between courses in Medicine and Nursing students, while García_Mangas [20] describes that the scores obtained in their questionnaire are worse in higher courses. The study of Tey [6] in nursing students only finds differences between first and last year courses, in the ethical aspects related to norms and rules. A study carried out in dentistry students [18] finds that there are not differences between courses regarding values of respect and responsibility, but there are differences in others such as participation and leadership.

The questions with the lowest scores corresponded to questions related to attitudes of solidarity and social participation. The majority of our students had little involvement in participatory processes either in the educational community or in social or political organizations. Neither these issues nor environmental responsibility was mentioned in the interviews of the qualitative phase, which is why we assumed that they were aspects that the students did not consider important at that time in their lives. In the interviews, the analysis of the student’s attitudes toward altruism indicated that altruism is associated with interaction with their companions and with patients and students did not consider altruism in a more social approach. This is similar to what Vicentela describes in his study [18], while in another study conducted in nursing students, it is described that 35% of students had volunteered and obtained a higher score on their questionnaire [6]. The authors of this study indicate that the age of nursing students is higher than the average of undergraduate students, which may explain their results.

### Limitations

Finally, it seems important to note some limitations this study had. For instance, as the sample comprised students from the courses that the researchers taught, it may be interesting in the future to collect a larger sample with participants from other courses in various Health Sciences degrees.

The interview question in the qualitative study was quite open. Accordingly, students could have benefited from instructions with a more specific timeframe. For example, the timeframe for first-year students should have referred to their high school experience instead of their college experience.

Other limitation is that the questionnaire explores the ethical perception of students in a general way. Given the importance that some specific professional ethical values have for the students of Health Sciences, we are designing a new tool to shed light on this new line of research.

Despite its limitations, we believe this questionnaire can be useful for teachers in the health area; not only analyzing students’ perceptions of ethical values, but also identifying areas for intervention or improvement. Students that demonstrate ethical competence will most likely be ethically responsible health professionals with key contributions to the development of their social/professional environment.

## Conclusions

Using a mixed research methodology, this investigation conducted an in-depth analysis of how Health Sciences undergraduate students self-perceive ethical values. In the initial qualitative phase, we noticed that the students were surprised when asked about ethical values, maybe because the subject matter is still unusual in the university context. For the quantitative analysis, our questionnaire results highlighted the ethical values of responsibility, the basic rules of education and respect as the most important values, whereas solidarity and social participation, some of the social aspects of ethical values, as the least important.

It may be of critical importance to disentangle the relationship of self-perception of ethical values and social desirability as the scores of our questionnaire were high in general and intimately related to social desirability, the more consistent and significant predictive variable in our multiple regression analyses.

The scores obtained on our questionnaire were correlated with the students’ age. This also entails that some educational interventions may be sensitive to some maturation processes related to the acquisition or development of ethical values. Future longitudinal studies should also focus on the progression of self-perception of ethical values in undergraduate and postgraduate students.

All in all, we believe this ad-hoc questionnaire and derived findings may be useful tools to college professors as well as other educators in the area of health with the aims of further analyzing the self-perception of their students’ ethical values and identifying the key areas for improvement/appraisal in their ethical competence.

## Additional files

Additional file 1: Questionnaire. Version of questionnaire designed during the study. (DOCX 27 kb)

Additional file 2: Raw dataset. Archive of data obtained in the study. (XLSX 104 kb)

## References

[1] Martínez M, Buxarrais MR, Esteban F (2002). La universidad como espacio de aprendizaje ético. Rev iberoam educ.

[2] Haigh M, Clifford VA (2011). Integral vision: a multi-perspective approach to the recognition of graduate attributes. HERD.

[3] Asalde R, Jara A, Flores M, Flores A, Fernandez E, Ñique C. Valores éticos compartidos entre profesionales de ciencias de la salud en el Perú. Flumen. 2016;7(2):5–66.

[4] ANECA. Guía de apoyo para la redacción, puesta en práctica y evaluación de los resultados del aprendizaje: ANECA; 2013 [01/03/2018]. Available from: http://www.aneca.es/content/download/12765/158329/file/learningoutcomes_v02.pdf.

[5] Bolívar A (2005). El lugar de la ética profesional en la formación universitaria. Rev Mex Inv Educ.

[6] Tey A, Vilà R, Martínez J (2014). Competencias para el aprendizaje ético en estudiantes universitarios de enfermería y pedagogía. Red U.

[7] Delors J (1996). La educación encierra un tesoro. Informe a la UNESCO de la Comisión Internacional sobre la Educación para el Siglo XXI.

[8] Boni A, Lozano JF (2007). The generic competences: an opportunity for ethical learning in the European convergence in higher education. J High Educ.

[9] García Retana JÁ (2011). Modelo educativo basado en competencias: importancia y necesidad. Rev actual investig educ.

[10] Esteban F, Buxarrais M (2004). El aprendizaje ético y la formación universitaria: mas allá de la casualidad [Ethical learning and university education: beyond chance]. Teor Educ.

[11] Morales FJ, Trianes Torres MV, Infante Cañete L (2013). Perfiles de valores éticos en estudiantes universitarios. Aula abierta.

[12] Deniz KZ, Türe E, Uysal A, Akar T. Investigation of vocational interest and preference in terms of gender and socio-economic status. EJER. 2014;(57):91–111.

[13] Yang Y, Barth JM (2015). Gender differences in STEM undergraduates' vocational interests: people–thing orientation and goal affordances. J Vocat Behav.

[14] Garcia R, Verde I, Vázquez V. ¿Por qué es necesario trabajar la dimensión ética en la docencia? XII Congreso Internacional de Teoría de la Educación; 2011; Barcelona.

[15] Díaz Flores M, Castro Ricalde DM, Cuevas Jaimes BL (2012). Valores profesionales de enfermería: Una mirada hacia la formación en la Educación Superior. Humanid méd.

[16] Montemurro D, Vescovo G, Negrello M, Frigo AC, Cirillo T, Picardi E (2013). Medical professional values and education: a survey on Italian students of the medical doctor school in medicine and surgery. N Am J Med Sci.

[17] Aguilar Martínez E, Anguiano Serrano SA, Coffin N (2014). Diferencias en la percepción de la adquisición de valores éticos en estudiantes de medicina y psicología de la FES Iztacala. REPI.

[18] Vicentela LA, Narváez CG, Velásquez M (2015). Valores éticos y formación curricular en odontología. Acta bioeth.

[19] Palomer L, López R (2016). Medición de los valores éticos y morales enseñados en la carrera de Odontología de la Pontificia Universidad Católica de Chile, desde la apreciación docente. FEM: Revista de la Fundación. Educación Médica.

[20] García-Mangas JA, García-Vigil JL, Lifshitz A (2016). Percepción de lo ético desde el punto de vista de los estudiantes de medicina. Rev Med Inst Mex Seguro Soc.

[21] García-Huidobro D, Núñez F, Vargas P, Astudillo S, Hitschfeld M, Gennero R (2006). Expectativas de estudiantes de medicina de pregrado en relación al perfil de médico esperado. Rev Med Chile.

[22] Amer Cuenca J, Martínez Gramage J (2009). Estudio del marco de referencia bioético en estudiantes españoles de fisioterapia. Rev iberoam fisioter kinesiol.

[23] Amador Guillermo L, Ortiz Villagómez G, Hernández Lomelí A, Ortiz Villagómez M, Alcocer Maldonado A, Hernández Montiel LH (2008). Perspectiva de los pacientes sobre los valores humanos en los profesores y estudiantes de licenciatura en odontología. Rev Odont Mex.

[24] Oseguera-Rodríguez J, Viniegra-Velázquez L (2008). Características humanistas del médico deseadas por la sociedad. Rev Med Inst Mex Seguro Soc.

[25] Puebla-Viera DC, Ramírez-Gutiérrez A, Ramos-Pichardo P, Moreno-Gómez MT (2009). Percepción del paciente de la atención otorgada por el personal de enfermería. Rev enferm Inst Mex Seguro Soc.

[26] Bazrafcan L, Nabeiei P, Shokrpour N, Moadab N (2015). Medical ethics as practiced by students, nurses and faculty members in Shiraz University of Medical Sciences. JAMP.

[27] Self DJ, Baldwin DC, Wolinsky FD (1996). Further exploration of the relationship between medical education and moral development. Camb Q Healthc Ethics.

[28] Hodelín Tablada R (2014). Fuentes Pelier D. El profesor universitario en la formación de valores éticos.

[29] Buxarrais MR, Esteban F, Mellen T (2014). The state of ethical learning of students in the Spanish university system: considerations for the European higher education area. HERD.

[30] Taylor S, Bogdan R. El trabajo con los datos. Análisis de los datos en la investlgacion cualitativa. Introducción a los métodos cualitativos de investigación, la búsqueda de significados. Barcelona: Paidós 1987. 152–174 p.

[31] Marlowe D, Crowne DP (1960). A new scale of social desirability independent of psychopathology. J Consult Psychol.

[32] Ferrando PJ, Chico E (2000). Adaptación y análisis psicométrico de la escala de deseabilidad social de Marlowe y Crowne. Psicothema.

[33] Denzin NK, Lincoln YS, Denzin NK, Lincoln YS (2000). The discipline and practice of qualitative research. Handbook of qualitative research.

[34] Pereira Z (2011). Los diseños de método mixto en la investigación en educación: Una experiencia concreta. Rev Electr Educare.

[35] Ugalde NU, Balbastre F (2013). Investigación cuantitativa e investigación cualitativa: buscando las ventajas de las diferentes metodologías de investigación. Cienc Econ (San Jose).

[36] Sánchez MC. La dicotomía cualitativo-cuantitativo: posibilidades de integración y diseños mixtos. Campo abierto. 2015:11–30.

[37] Bisquerra R. Metodología de la Investigación Educativa. 2 ed. Madrid: La Muralla; 2004.

[38] Gervilla CE (2000). Un modelo axiológico de educación integral. Rev Esp Pedagog.

[39] Gómez Padrón EI, Morales SI. Fundamentos para la evaluación cualitativa de la formación de valores en carreras de la salud. Rev Cuba Educ Méd Super. 2009;23(3):70–81.

[40] Pérez Quiñones JA, Hernández Falcón L, García García LE, Rodríguez C, del Carmen M, Hernández Díaz O (2014). Importancia de la orientación educativa en la formación de valores en las universidades de Ciencias Médicas. Revista Médica Electrónica.

[41] Singh L, Espinosa M, Columbié N, Cantillo Y (2017). Valores ético-morales y calidad de los servicios prestados de enfermería en Guantánamo. RIC.

[42] Nunes R, Duarte I, Santos C, Rego G (2015). Education for values and bioethics. Springerplus.

[43] Salazar VF, Rodríguez CAF (2016). Valores éticos en la formación del estudiante de Psicología en la UAS/ethical values in the formation of psychology students at the Autonomous University of Sinaloa. Rev Iberoam Cienc Soc Humaníst.

[44] Larios GE. Educación en valores. RAITES. 2017;3(6):69–87.

[45] Leal B (2006). Moral values in the Modern University: the search for a positive contribution: a REVIEW OF. HERD..

[46] Navarro G, Cottin I, Fasce E, Pérez C (2009). Valores y orientación social en estudiantes de medicina de primero y séptimo año de la Universidad de Concepción. Rev Educ Cienc Salud.

[47] Grootenboer P (2010). Affective development in university education. HERD.

[48] Mercader V (2006). Study of the ethical values of college students.

[49] Capote E, Alejandra BM, Natacha G, Villegas H, Capote J (2006). Comparación de la jerarquía de valores entre los estudiantes de medicina y odontología del segundo año de la universidad de carabobo. Acta Odontol Venez.

[50] Grieve R, McSwiggan C (2014). Predicting intentions to fake in psychological testing: which normative beliefs are important?. Eur J Work Organ Psychol.

[51] Nordholm LA (1974). A note on the reliability and validity of the Marlowe-Crowne scale of social desirability. J Soc Clin Psychol.

[52] Crino MD, Svoboda M, Rubenfeld S, White MC (1983). Data on the Marlowe-Crowne and Edwards social desirability scales. Psychol Rep.

[53] Tanaka-Matsumi J, Kameoka VA (1986). Reliabilities and concurrent validities of popular self-report measures of depression, anxiety, and social desirability. J Consult Clin Psychol.

[54] Holden RR, Fekken GC (1989). Three common social desirability scales: friends, acquaintances, or strangers?. J Res Pers.

[55] Moral de la Rubia J, García CH, Antona CJ (2012). Traducción y validación del Inventario Balanceado de Deseabilidad Social al Responder en una muestra probabilística de estudiantes universitarios mexicanos. Revista de Psicología GEPU.

[56] Montilva M, García M, Torres A, Puerta M, Zapata E (2015). Empatia en estudiantes venezolanos de medicina y enfermeria según género, nivel de la carrera y antecedentes de hospitalizacion. Revlatinoambioet.

