# Supplementary material for: Ethical values in college education: a mixed-methods pilot study to assess health sciences students’ perceptions
Source: BMC Med Educ. 2018 Dec 4;18:289. doi: 10.1186/s12909-018-1396-7 (PMC6278160; doi:10.1186/s12909-018-1396-7)
Supplement: Supplementary file 1 — Questionnaire. Version of questionnaire designed during the study. (DOCX 27 kb) [file 12909_2018_1396_MOESM1_ESM.docx]

*Dear Student,*

*We request your collaboration in an educational research project that aims to "identify the aspects related to the values and ethical commitments that students of Health Sciences consider most important".*

*If you are interested in collaborating in this project, you must complete the following information and answer anonymously the questions of the attached questionnaire.*

*Thank you very much for your help.*

AGE: MALE FEMALE

TITULATION: ___________________________________ COURSE: _________

| 1 Totally disagree  2 Somewhat disagree  3 Somewhat agree  4 Totally agree |
| --- |

*Mark with a cross in the box that corresponds to your degree of agreement or disagreement with the statements that appear below.*

*Answer with sincerity. There are not correct or incorrect answers.*

|  | 1 | 2 | 3 | 4 |
| --- | --- | --- | --- | --- |
| 1. I am responsible for my actions |  |  |  |  |
| 1. After a conversation, I can change my point of view |  |  |  |  |
| 1. I know the standards of the university and my degree. |  |  |  |  |
| 1. My manner of acting with others is consistent with my ideas |  |  |  |  |
| 1. I respect my environment, I do not litter, and I do not deface urban surfaces |  |  |  |  |
| 1. When I fail a subject, I can assess what I can do to improve |  |  |  |  |
| 1. I like to be updated when there is important news to reflect on what has occurred |  |  |  |  |
| 1. If a teacher makes fun of a co-worker, I let my coordinator/tutor/delegate know about it |  |  |  |  |
|  | 1 | 2 | 3 | 4 |
| 1. When someone is speaking, I do not generally interrupt, and I wait for him/her to finish |  |  |  |  |
| 1. It seems bad to me when someone insults or interrupts a classmate or teacher |  |  |  |  |
| 1. I turn to dialogue as a strategy to address conflicts |  |  |  |  |
| 1. I have an open attitude and close relationships with my colleagues |  |  |  |  |
| 1. I use trash bins appropriate to each type of waste |  |  |  |  |
| 1. When I use computers at the university, I turn them off when I finish using them |  |  |  |  |
| 1. I take special care with the material provided to me by the university (laboratories, classrooms, stretchers, tables, etc.) |  |  |  |  |
|  | 1 | 2 | 3 | 4 |
| 1. I am aware that consensus/agreement is not always reached through dialogue |  |  |  |  |
| 1. If a friend becomes angry with me, I reflect on his reaction to try to understand him/her. |  |  |  |  |
| 1. I know how to be in someone else´s shoes |  |  |  |  |
| 1. I introduce myself or participate in the election of delegates of courses |  |  |  |  |
| 1. If a teacher suspends me unjustly, I know how to control my anger |  |  |  |  |
| 1. I give up my seat on the bus to elderly, pregnant or disabled persons |  |  |  |  |
|  | 1 | 2 | 3 | 4 |
| 1. I am able to defend my opinion about political or religious news although my colleagues may think otherwise |  |  |  |  |
| 1. When a group of colleagues unfairly criticizes a friend of mine, I position myself in his/her favor |  |  |  |  |
| 1. It is important to have a course delegate to speak to teachers and the university |  |  |  |  |
| 1. I know how to organize my study and leisure hours |  |  |  |  |
| 1. When a colleague speaks to me during a teacher’s explanation, I ask him/her to let me pay attention |  |  |  |  |
|  | 1 | 2 | 3 | 4 |
| 1. I base my opinions on reasoned arguments |  |  |  |  |
| 1. I favor a good group environment in my work team |  |  |  |  |
| 1. I participated in a group or organization of a social or political nature |  |  |  |  |
| 1. I know how to explain my opinions when a topic of discussion emerges in my group of friends |  |  |  |  |
| 1. When a colleague thinks differently from me, I attempt to respect his/her opinion. |  |  |  |  |
| 1. When a course starts, I make sure to read the program and the standards |  |  |  |  |
| 1. I show my disapproval of unfair treatment to a person of a different race |  |  |  |  |
|  | 1 | 2 | 3 | 4 |
| 1. I relate to people of different religious beliefs, respecting their convictions |  |  |  |  |
| 1. I recognize good arguments even if they do not coincide with my own |  |  |  |  |
| 1. If there is a conflict between colleagues, I insist that we listen to one another and reach an agreement |  |  |  |  |
| 1. I read the news at least once a week |  |  |  |  |
| 1. I am involved in a volunteer activity |  |  |  |  |
| 1. I reuse products that I consume as much as possible |  |  |  |  |

THANK YOU VERY MUCH FOR YOUR COOPERATION
